# Communication strategies used in pediatric urology by specialist professionals

**DOI:** 10.1590/0034-7167-2025-0325

**Published:** 2026-07-31

**Authors:** Cristiane Feitosa Salviano, Gisele Martins

**Affiliations:** I Hospital da Criança de Brasília José Alencar. Brasília, Distrito Federal, Brazil; II Universidade de Brasília. Brasília, Distrito Federal, Brazil

**Keywords:** Health Communication, Child Language, Urology, Child, Health Personnel., Comunicación en Salud, Lenguaje Infantil, Urología, Niño, Personal de Salud.

## Abstract

**Objectives:**

to describe the communication strategies used by urology specialists to obtain information directly from children.

**Methods:**

a qualitative study with professionals who had been treating children with urinary and intestinal symptoms for at least one year. Semi-structured interviews were carried out, and thematic analysis was conducted and mediated by NVIVO version 12.0.

**Results:**

fourteen specialist professionals were interviewed. From their statements, three thematic categories emerged: (1) Verbal communication, with adaptation of language, use of simple terms and analogies; (2) Nonverbal communication, with visual resources such as drawings, scales and voiding diaries; (3) Play and playful strategies, such as the use of dolls to demonstrate positioning on the toilet, dramatizations and games to facilitate verbal expression of symptoms.

**Final Considerations:**

language adaptation, photo-elicitation, and playful tools prove to be effective communication strategies within pediatric urology. These strategies are low-cost, and can be developed and adapted to clinical practice.

## RESUMEN

**Objetivos:**

describir las estrategias de comunicación utilizadas por los especialistas en urología para obtener información directamente de los niños.

**Métodos:**

estudio cualitativo con profesionales que habían tratado a niños con síntomas urinarios e intestinales durante al menos un año. Se realizaron entrevistas semiestructuradas y se realizó un análisis temático mediado por NVIVO, versión 12.0.

**Resultados:**

se entrevistó a 14 profesionales especialistas. De sus declaraciones, surgieron tres categorías temáticas: (1) Comunicación verbal, con adaptación del lenguaje, uso de términos sencillos y analogías; (2) Comunicación no verbal, con recursos visuales como dibujos, escalas y diarios miccionales; (3) Juego y estrategias lúdicas, como el uso de muñecos para mostrar la posición en el inodoro, dramatizaciones y juegos para facilitar la expresión verbal de los síntomas.

**Consideraciones Finales:**

la adaptación del lenguaje, la fotoelicitación y las herramientas lúdicas resultan ser estrategias de comunicación eficaces en urología pediátrica. Estas estrategias son económicas y pueden desarrollarse y adaptarse a la práctica clínica.

## INTRODUCTION

Effective communication in pediatric urology is fundamental to the diagnostic process and therapeutic adherence. Diagnosing urological conditions requires gathering information based on reports of elimination and lifestyle habits, such as urinary and fecal frequency, physical aspects of urine and feces eliminated, urinary stream, elimination behaviors (delaying urination and/or defecation, holding maneuvers), dietary and fluid habits, among others^(1,2)^. Both diagnosis and treatment are based on the child’s and family’s reports. Therapeutic adherence involves self-management strategies implemented by the child and family, supported by a specialized professional, with communication being a constant mediator in this process. Regarding therapeutic options, standard urotherapy stands out as a first-line treatment for functional disorders, such as lower urinary tract dysfunctions. Composed of five components, standard urotherapy includes information and demystification, modification of voiding and bowel habits, lifestyle counseling, recording of symptoms and voiding habits, and finally, support and encouragement^(2,3)^.

In this context, even with the possibility of gathering information from primary caregivers, children are an excellent source of health information, requiring communication strategies adapted to their age group. Giving children an active voice within healthcare settings is challenging and rarely implemented. In an analysis conducted, children between 5 and 11 years old mostly stated that professionals did not even introduce themselves before starting care in an inpatient setting, and 72.70% of this age group reported not understanding the health information provided, reflecting an inadequate communication strategy for this age group^(4)^. This type of professional conduct restricts the children’s right to be informed about their health in appropriate language and to have their doubts and fears addressed by a healthcare professional^(5)^.

Communication with children requires multiple factors to be clear and effective. Professionals’ communication skills, caregivers’ involvement in the exchange of information, and the use of communication strategies that adapt the message to recipients’ cognitive development level are examples of fundamental elements for successful communication between professional and child^(4)^. In this context, it is observed that healthcare professionals need internal skills and communication tools for effective interaction. Communication skills begin during professional training and are honed through practice, while tools are often developed on-the-job and end up being restricted to the workplace.

As a way to expand communication tools in pediatrics, the literature is growing with examples of strategies that use technology as a mediator in various pediatric specialties^(6-8)^. Some professionals make use of low-tech methods, such as booklets, dolls^(9)^, therapeutic toys^(10)^, among others. However, the literature is scarce regarding specific cases and strategies in pediatric urology based on solid theoretical frameworks, with the common use of empirically based strategies. Such practices are not widely disseminated and, in many cases, are only shared within the healthcare service where professionals work.

## OBJECTIVES

To describe the communication strategies used by professionals specializing in pediatric urology to gather information directly from children.

## METHODS

### Ethical aspects

The study was conducted in accordance with national and international ethical guidelines and approved by the Research Ethics Committee of the School of Health Sciences at the *Universidade de Brasília*. Considering the ethical standards for research involving human subjects, the application of a semi-structured instrument was preceded by the signing of an Informed Consent Form and an Authorization Form for the Use of Image and Voice Recordings for research purposes. In relation to the identification of the different participants, the designations PF1, PF2... PF14 were used in order to preserve professionals’ anonymity.

### Study theoretical and methodological framework and design

This is a descriptive study, with a qualitative approach, carried out with specialist/expert professionals working in the field of pediatric urology in Brasília. The study is part of a doctoral thesis research project entitled “*Experiência de crianças com sintomas urinários e intestinais: contando essa história*”^(11)^. The methodological description followed the Consolidated criteria for Reporting Qualitative research checklist^(12)^.

### Study setting

The research was developed in the Federal District (FD), in the outpatient unit of a public teaching hospital, as well as two other health units - one private and the other public from the Brazilian Unified Health System of the FD -, with the aim of ensuring the participation of a diversity of specialized professionals.

### Data source

The sample was one of convenience, and the snowball sampling technique was used to identify professionals. The sample was selected from professionals with expertise in pediatric urology and at least one year of experience in treating children with urinary and/or intestinal symptoms. The number of professionals included in the sample followed the data saturation criterion^(13)^. The process was monitored during the thematic analysis through continuous comparison between interviews and coding matrix tracking. Saturation was considered reached when statements began to show recurring meanings and no new conceptual elements emerged. The final decision was validated by consensus among the researchers responsible for the analysis.

### Data collection and organization

Data collection took place between February and May 2019. The data were collected by a nursing student researcher, previously trained in interview techniques. Interviews were conducted in the professionals’ workplace at scheduled times to avoid disrupting their work routine. The guiding questions were: when investigating urinary and bowel symptoms, do you usually interview/talk to the child, the caregiver, or both? How do you conduct this investigation? What communication strategies do you use with children?

The individual interviews lasted an average of 12 minutes. Of the 14 professionals approached and invited to participate in the research, all promptly accepted. There were no refusals, and they were informed about the purpose of the research during the initial contact.

### Data analysis

The interviews were audio-recorded and transcribed, and analyzed using NVIVO version 12.0. Field notes were also taken after each interview, and it was not necessary to re-contact any professional for confirmation or clarification of the analyzed content.

The data analysis method followed the stages proposed by Braun and Clarke^(14)^, which included the following stages: data familiarization, through transcription and exhaustive reading of interviews; initial code production; topic exploration; topic review; topic definition and naming; and report production. All stages were carried out with the aid of the NVIVO program and with the verification of consensus among the researchers involved in the data analysis process.

## RESULTS

The sample consisted of 14 specialist professionals from the following categories: nurses (n=4); physicians (n=5); and physiotherapists (n=5). The mean professional experience was seven years and 11 months (ranging from two to 25 years). All nurses were currently enrolled in postgraduate courses, master’s programs, and/or residencies in pediatric nursing, specifically in pediatric urology or nephrology. All physicians had completed residencies in pediatric nephrology. Physiotherapists had specializations in pelvic dysfunction, pelvic floor rehabilitation, urogynecological physiotherapy, pelvic physiotherapy, or had obtained master’s and/or doctoral degrees in medical sciences with dissertations in the field.

**Figure 1:**
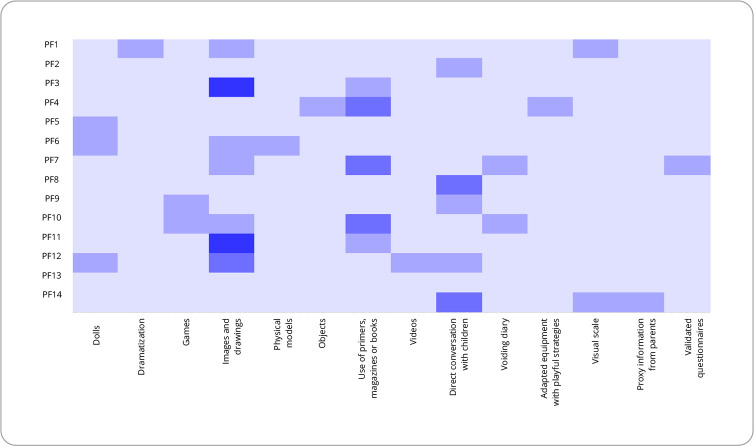
Data coding matrix, Brasília, Distrito Federal, Brazil

The data from the interview were analyzed and coded, primarily composing the coding matrix (Figure 1). This matrix illustrates the initial data organization process, in which codes emerged from professionals’ statements. Shades indicate the frequency of occurrence of codes in the interview, with darker shades signaling greater recurrence.

**Figure 2:**
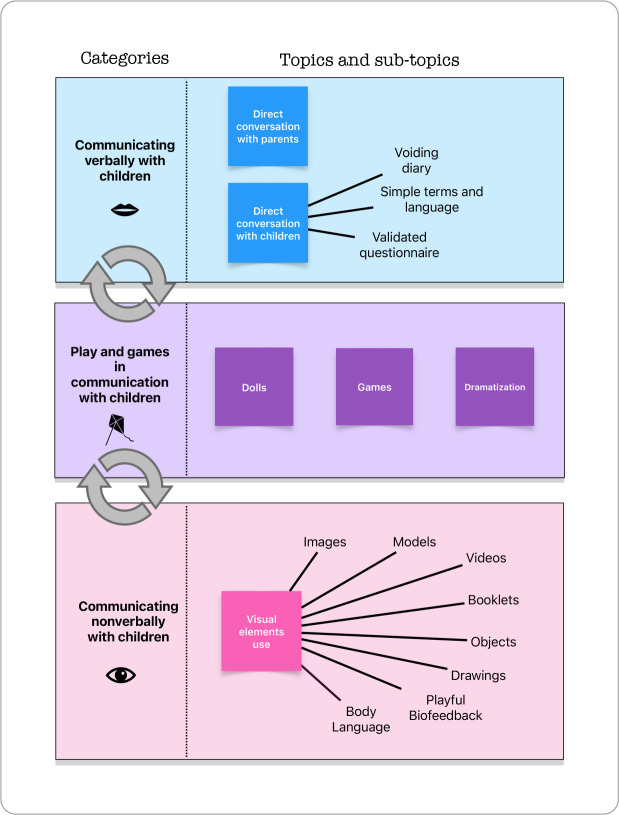
Themed tree, Brasília, Distrito Federal, Brazil

This thematic tree (Figure 2) presents the consolidation of codes into three main categories: Verbal communication; Nonverbal communication; and Play strategies. The diagram highlights how the categories interrelate, demonstrating that nonverbal communication often complements verbal communication, while play integrates elements of both, configuring itself as a hybrid strategy. This visual representation allows us to understand the analytical path, from the initial coding to the formation of categories that reflect concrete practices of interaction with children in the context of pediatric urology.

### Communicating verbally with children

Verbal communication is the language format described in this thematic category. The healthcare professionals interviewed describe how they conduct their care and divide their perceptions between conversations directed at primary caregivers/parents and directly to children.

Dialogue with caregivers/parents proves to be the most traditional and comfortable approach for both professionals and primary caregivers/parents. It was commonly observed that caregivers take the lead in the dialogue, preventing children from taking the lead. This is a frequently used method for gathering health information about the children being cared for.

*Often, mothers don’t even let the children speak, but I try to get something from the children first. The mothers add to it - of course they’ll give more information, but I try to get something out of them, some insight.* (PF1)

Professionals also demonstrated how to conduct conversations directly with children. Their main strategy is the use of simple language and terms to address health issues, as well as the frequent use of diminutive terms to adapt the message to children’s level of understanding. Analogies were also implemented in the dialogues, such as associating the bladder with the “pee house”.

*The main issue is communication; it’s about sitting down with children and trying to communicate effectively* [...] *you’re willing to listen to them and try to speak in a language they understand. You have to make yourself understood by the child in order to pick up on these symptoms.* (PF6)

*Well* [...] *I talk directly to them* [the children], *so the consultation doesn’t become boring, so they don’t think it’s something the mother has to come and talk to me about* [...] *I try to use simpler language, and even if it’s a small child, after the father tells me, I always go back and ask the child again.* (PF7)

*Maintaining language that’s also more appropriate for the child so they don’t use overly difficult terms* [...] *that they understand. We use “pee” and “poop”, “tummy ache”, “ran away”, “pee wet her panties”.* (PF3)

*So, the expressions we use, I always bring up “bladder” and “intestine”, because I need to say that it’s the place for pee, the place for poop. And I don’t talk about the anal canal and urethra, I’m going to talk about the “little door”, the little door of the intestine, the little door of the bladder.* (PF10)

Professionals also used analogies from the child’s daily life to guide the conversation, bringing the listener closer to the message being conveyed.

*So, the child asks, “Why do I have to drink so much water?” and then you could explain the whole physiological functioning of the body and everything else, or you could give a simpler example, a much simpler analogy that they usually understand quite well. So, “Don’t you bathe every day?”, “Don’t you wash your body every day?”. So, you have to wash your body inside too. There you go, then they understand, it becomes much easier, much more relaxed.* (PF4)

*Well* [...] *I try to bring them down to earth by asking about this issue* [...] *“If you’re going to sleep over at a friend’s house and you wet the bed, are you embarrassed?”, “Are you going to your friend’s house or not?”, to see how much this hinders the child’s social development.* (PF2)

*I always try to bring them what they can* [...] *in a way they can understand* [...] *within their routine, you know* [...] *within what they are able to understand.* (PF5)

Validated clinical diagnostic tools are also cited by interviewees as strategies that aid the communication process. The Bristol Stool Scale and the voiding diary guided the dialogues and gathered information from both the child and the primary caregivers.

*I usually show them the Bristol Stool Scale. The children laugh and are sometimes embarrassed, or I ask, “What does your poop look like?” and then I show them the picture.* (PF1)

*I usually get that Bristol stool scale from the computer, you know, the one for feces.* (PF14)

*So, here we use* [...] *uh* [...] *we have a little diary, you know* [...] *we keep a diary. We do some quality of life questionnaires so we can see if we can* [...] *uh* [...] *better capture the patient’s information.* (PF8)

*So, I always try to show them what will happen within their own reality. “So, we’re going to keep this diary here, but this diary is to know if you’re peeing at the right time, so this clock that we’re going to use is to help you remember when you have to pee”.* (PF5)

### Communicating nonverbally with children

Unlike the first thematic category, this section addresses strategies that utilize nonverbal language to guide communication with children. Images and drawings were the most frequently cited strategies by professionals in this area. Their use was based on the principles of employing playfulness in communication, as well as seeking to materialize examples, making them more concrete at the children’s level of thought. Professionals used this strategy both for the expression of feelings and emotions and for the physical representation of the human body, which facilitates the identification of symptoms and a more concrete exemplification of positions, for instance.

*I use drawings of the bladder, kidneys, and ureters to explain how the bladder fills and empties.* (PF3)

*Trying to be as visual as possible* [...] *is* [...] *showing an image. So, I have a little magazine that I carry in my bag with me. It shows the abdominal area, and then, there in the abdominal area, I start by saying, “Ah, if you remove this part of the skin, this is what you’ll see”, “If you touch it, look, touch it here”, “Do you know where, when you feel the urge to pee, where it hurts?” Then they point, right? They point. They know where the bladder is. “So, the bladder is this, it’s a little balloon here”.* (PF4)

*I always bring, sometimes, a drawing, and I ask them to draw how they feel, so that they understand what’s happening, and then I show them through the drawings themselves. “Look, in this case, you have a little problem here”.* (PF5)

*And drawings, lots of drawings, showing her* [...] *making that information tangible for the child. Since she’s very visual and playful, we always try to show her something so she can associate that information with the object she saw.* (PF9)

*I also demonstrate* [...] *I use the computer to show those compression and squatting techniques to the parents and the child, to see if they recognize them as their own behavior, you know?* (PF14)

Other examples of tools used are brochures, magazines, and books. Professionals state that these materials contain important information about treatment, presenting images that support the illustration of clinical guidelines and allowing children to identify with their own experience of symptoms. Thus, such health education resources also play a significant role in communicating with children.

*I use brochures so that they can also see, through images and drawings, the little booklets - more geared towards the parents, which they will read, and for the child who already knows how to read, we give them to the child.* (PF3)

*If it’s a very difficult child, they don’t really know how to talk about or report their symptoms. We use a lot of educational books, especially for those little children who are in the potty training phase, things like that.* (PF8)

*I have a little magazine that I carry in my briefcase. It will show the abdominal area.* (PF4)

*I use books related to the topics.* (PF11)

*I also have a little magazine that shows that it’s common for children to wet the bed while sleeping, and that it’s not just a problem for them; it can happen to many children. It’s a fun magazine that I bring to them and show them how important it is to follow the treatment correctly so that they can see results and improve in that regard.* (PF5)

Less frequently mentioned in the interviews were models, videos, and everyday objects. All of these serve the purpose of making the information more tangible, within a perspective of a concrete message.

*We have a dynamic model there that I use when I’m explaining the urinary system. It’s really cool, really big* [gestures]*; it has the whole urinary system. So, I explain it to them... and it catches their attention.* (PF9)

*So that they can see and visualize it, I also use video.* (PF3)

*I use a cup to make the sound of peeing. Anyway, anything playful we can think of, you know* [...]. (PF11)

Even neuromodulation therapy devices, such as biofeedbackers, are adapted with images to aid communication with children.

*In short, we use everything playful that we have, right* [...] *playful biofeedback, right?* (PF11)

### Play and games in communication with children

This category includes play and games as communication tools. It is, therefore, a category that encompasses verbal and non-verbal elements, i.e., fundamental parts of play, and is a category that relates to the first two.

The use of dolls was one of the play strategies adopted. The doll allows for the demonstration of the urinary and intestinal systems, and children’s identification with the symptoms they present. Furthermore, professionals use the doll to illustrate clinical guidelines, such as positioning on the toilet. It should be remembered that these are not necessarily specific urology dolls, but often adapted to the guidelines and context, with felt being one example of a material used for doll construction.

*When I go, for instance, to* [...]*demonstrate the correct toilet positioning, we have two large cloth dolls there - a girl and a boy. When it’s a girl, I take a girl doll and try to show her, making the doll position itself correctly* [...] *“Look, your feet have to be flat on the floor, right, so you can relax your pelvic floor”* [...] *and I explain* [...] *of course, in child-friendly language.* (PF9)

*We have a felt doll that has a bladder and intestines because, sometimes, when I’m dealing with these expressions, it’s easier for children aged 6 to 12 when I talk about bladders and intestines.* (PF10)

Storytelling was also a playful strategy adopted. Professionals used dramatization and demonstration to ensure the child had a direct understanding of the clinical guidance.

*I do theater. I myself show that the child has to bend down, especially the girl, open her legs, put her foot down, then I teach her that she has to sit on the toilet with her legs* [...] *with her knees wide apart. When, especially, the girls forget, etc., I tell them that they have to sit backwards on the toilet because of the shape of the toilet. If you go in facing forward, looking at the flush, there’s no way you won’t open your legs.* (PF14)

Games and toys have also been allies in the communication process, especially for reinforcing guidance and capturing symptoms experienced by children. It is important to emphasize that they are also produced by the professionals themselves, since there are not many options available on the market.

*I have some memory games that I sometimes show them.* (PF5)

*We use some toys, some games, so they can try to express these symptoms they have.* (PF6)

## DISCUSSION

Verbal communication is the most traditional form of communication, and this is no different in the context of pediatric urology care. Although consultations are mostly directed at caregivers^(4)^, interviewees offered suggestions for conducting direct communication with children. They based their suggestions on knowledge about child development and demonstrated that the conversation should be appropriate to the children’s developmental level. They adapt the language, seeking similarities with everyday life to make the subject matter more palatable. The topics to be addressed by professionals can be complex, such as demystifying the urinary and intestinal systems as part of urotherapy^(15)^.

Adapting language, such as using simple terms and diminutives, proved essential in bringing professionals closer to children’s cognitive and emotional world. This approach aligns with Piaget’s theoretical model, which describes the concrete operational stage (7 to 11 years) as a period in which children’s thinking is predominantly concrete and based on direct experiences with reality^(16)^. In this context, children’s symbolic thinking is characterized by the use of images and concrete representations of their daily experiences^(17)^. By employing communication that resonates with children’s cognitive perspective, professionals not only facilitate understanding but also create a more welcoming and safer environment, fostering spontaneous expression and children’s active engagement in dialogue.

Considering that urinary and bowel symptoms are more frequent in schoolchildren^(1,18)^, from Piaget’s perspective, it is justified that communication strategies should be fundamentally based on concrete thinking, both in verbal and non-verbal techniques. The use of images, videos, booklets, among others, was cited as a way to materialize the guidelines given by professionals, since they need to work with information and demystification about the symptoms and systems that make up urination and defecation, promoting the modification of urination and defecation habits - elements of urotherapy^(2,15)^. Similar experiences in pediatric nursing demonstrate that the use of visual materials and role-playing facilitates the understanding of physiological processes, encouraging children’s active participation in their care^(19,20)^. Thus, the use of concrete and playful resources contributes to the effectiveness of educational interventions in pediatric urology, reinforcing the role of nursing in mediating learning and therapeutic adherence.

These methods are already employed in other areas of health^(21-23)^, such as the use of imaging for pain localization^(24)^, demonstrating potential application in pediatric urology, especially in supporting communication and health education. A study on how children express their interests during hospitalizations highlights the provision of preparatory information for decision-making as a suggestion, which includes the use of images that represent the procedures to which children will be subjected^(25)^. International studies, mapped in a scoping review, on pediatric urinary incontinence also highlight the potential of digital tools and animated videos in children’s education, with results similar to those obtained by standard urotherapy in reducing urinary and bowel symptoms^(26)^. For nursing, these findings indicate opportunities to develop and validate audiovisual resources that integrate technical language and child sensitivity, strengthening educational practice and therapeutic adherence.

Arts-based approaches also align with the points made by interviewees, since the examples presented can be classified into types of art, such as drawings, videos, booklets (visual arts), dolls and dramatization (performing arts). The arts are widely used in interaction with children by pedagogy and psychology^(27-29)^, in health education actions^(19,20,22,23)^, and in pediatric research for accurate data collection^(30-32)^.

The use of play and therapeutic dolls aligns verbal and non-verbal language within a storytelling context. Storytelling is a form of communication widely accepted by children, who, through mirror neurons, are able to project themselves into the story, better understanding the messages and externalizing their own experiences^(33)^. The therapeutic felt doll mentioned by professionals is primarily used for health education. Furthermore, the literature shows other uses for the doll, including the expression of feelings related to treatment, the simulation of clinical procedures, and the training of physiological functions. The benefits of this type of technique are well-known, noted not only by the healthcare team but also by family members^(34)^.

In addition to the use of dolls, initiatives that combine art, history, and technology in pediatric urology are possible and suit children’s current preference for technological play. For instance, a recent prototype of a digital story aimed at serving these children can be cited^(11)^. Other examples of the use of technology in urotherapy are applications. Although not mentioned by interviewees, it is possible to identify them for incontinence^(35,36)^, including formats that act as a therapeutic intervention by providing reminders to patients and as a health education tool^(36)^, as well as applications that assist in monitoring urinary frequencies and facilitate the voiding diary completion^(37)^. Despite the many app options available, few are aimed at children.

### Study limitations

Two main limitations of the study can be considered. The first concerns the regionalization of data collection, since communication is also impacted by region and culture. The second relates to work settings. As professionals were identified using the snowball sampling technique, there was a predominance of three healthcare services. Nurses, for instance, were trained in the same clinic, and therefore, the strategies indicated by interviewees were similar.

Therefore, future studies should expand the number of healthcare services and extend data collection to other Brazilian states in order to identify specific and regional communication strategies. Despite the limitations, Brasília is a multicultural city and has historically received many migrants from different regions, which may have minimized the study’s limitations. The services where the interviewed professionals work have a high volume of patient care in the city, potentially reflecting a broader overview. Furthermore, the qualitative design allows for a more in-depth look at communication strategies, exploring the studied phenomenon with greater depth.

### Contributions to nursing, health, or public policy

The conclusions of this study highlight the critical need to improve pediatric urology healthcare professionals’ communication skills. The training and continuing education of these professionals should promote the development of specific skills to interact effectively with children and their family, especially in the context of complex interventions. This training should encompass not only verbal communication but also the use of nonverbal and playful strategies that foster understanding, therapeutic adherence, and the professional-child/family bond.

For clinical practice, it is suggested that it is important to systematically integrate approaches that combine these three categories of communication in the care of children in pediatric urology. The implementation of these strategies, which are generally low-cost and easy to apply, can significantly improve quality of care and children’s experience in healthcare settings.

## FINAL CONSIDERATIONS

Different communication strategies for gathering information directly from children were presented by the professionals interviewed and specialists in pediatric urology. It was possible to classify the strategies as verbal, non-verbal, and playful. However, it is evident that professionals do not use only one type of strategy, and it is common to combine all three categories in a single consultation.

Thus, language adaptation, photo-elicitation, and contextualization of guidelines with playful tools prove to be effective communication models within pediatric urology. These findings provide support for the construction of urological health education tools, the development of specific communication protocols for pediatrics, and the planning of professional training actions aimed at developing communication skills.

Thus, the study provides examples of low-cost and easily implemented strategies for daily care, contributing to improved interaction between professionals and children in pediatric urology, promoting greater adherence to treatment and potentially improving clinical outcomes. Furthermore, it highlights opportunities for the development and validation of new communication tools in future research that can be widely incorporated into clinical practice and assessed through longitudinal studies.

## Data Availability

The research data are available within the article.
