# Supplementary material for: Communication strategies used in pediatric urology by specialist professionals
Source: Rev Bras Enferm. 2026 Jul 31;79:e20250325. doi: 10.1590/0034-7167-2025-0325 (PMC13435855; doi:10.1590/0034-7167-2025-0325)
Supplement: Translation

# Translation: Estratégias comunicacionais utilizadas em urologia pediátrica por profissionais especialistas

**DOI:** 10.1590/0034-7167-2025-0325pt

**Authors:** Cristiane Feitosa Salviano, Gisele Martins

**Affiliations:** I Hospital da Criança de Brasília José Alencar. Brasília, Distrito Federal, Brasil; II Universidade de Brasília. Brasília, Distrito Federal, Brasil

**Keywords:** Comunicação em Saúde, Linguagem Infantil, Urologia, Criança, Pessoal de Saúde.

## Abstract

**Objetivos:**

descrever as estratégias de comunicação utilizadas por profissionais especialistas em urologia para captar informações diretamente da criança.

**Métodos:**

estudo qualitativo com profissionais que atendiam crianças com sintomas urinários e intestinais há pelo menos um ano. Entrevistas semiestruturadas foram realizadas, e análise temática foi conduzida e mediada pelo NVIVO, versão 12.0.

**Resultados:**

entrevistaram-se 14 profissionais especialistas. A partir de suas falas, emergiram três categorias temáticas: (1) Comunicação verbal, com adaptação da linguagem, uso de termos simples e analogias; (2) Comunicação não verbal, com recursos visuais como desenhos, escalas e diários miccionais; (3) Brincadeiras e estratégias lúdicas, como o uso de bonecos para demonstrar posicionamento no vaso sanitário, dramatizações e jogos para facilitar a expressão verbal de sintomas.

**Considerações Finais:**

adaptação da linguagem, foto-elicitação e ferramentas lúdicas mostram ser estratégias efetivas de comunicação dentro da urologia pediátrica. Tais estratégias são de baixo custo e podem ser desenvolvidas, adaptando-as à prática clínica.

## INTRODUÇÃO

A comunicação efetiva na urologia pediátrica é fundamental para o processo diagnóstico e adesão terapêutica. O diagnóstico de condições urológicas requer a captação de informações com base no relato de hábitos de eliminação e de vida, tais como frequência miccional e fecal, aspectos físicos de urina e fezes eliminadas, jato miccional, comportamentos de eliminações (ato de postergar micção e/ou evacuação, manobras de contenção), hábitos alimentares e hídricos, entre outros^(1,2)^. Tanto o diagnóstico quanto o tratamento se baseiam no relato da criança e da família. A adesão terapêutica perpassa estratégias de automanejo que são implementadas pela criança e família, apoiadas pelo profissional especializado, sendo a comunicação uma mediadora constante desse processo. Quanto às possibilidades terapêuticas, destaca-se a uroterapia padrão como primeira linha de tratamento para desordens funcionais, como as disfunções do trato urinário inferior. Composta por cinco componentes, a uroterapia padrão inclui informação e desmistificação, modificação de hábitos miccionais e de evacuação, aconselhamento de estilo de vida, registro de sintomas e hábitos miccionais, e, por último, o suporte e encorajamento^(2,3)^.

Nesse contexto, mesmo com a possibilidade de captar informação dos cuidadores primários, a criança é uma excelente fonte de informação em saúde, requerendo estratégias de comunicação adaptadas à sua faixa etária. Proporcionar uma voz ativa à criança dentro do cenário de atendimento em saúde é desafiador e pouco implementado. Em análise realizada, crianças entre 5 e 11 anos afirmaram, em sua maioria, que os profissionais sequer se apresentavam antes de iniciar cuidados em cenário de internação, e 72,70% desse grupo etário informou não compreender as informações de saúde prestadas, retratando uma estratégia de comunicação inadequada para a faixa etária^(4)^. Esse tipo de postura profissional cerceia o direito da criança de ser informada sobre sua saúde em uma linguagem adequada e de ter suas dúvidas e receios sanados pelo profissional de saúde^(5)^.

A comunicação com a criança requer múltiplos fatores para que seja clara e de qualidade. A habilidade de comunicação do profissional, o envolvimento dos cuidadores na interlocução da informação e o uso de estratégias comunicacionais que adequem a mensagem ao nível de desenvolvimento cognitivo do receptor são exemplos de elementos fundamentais para o sucesso da intercomunicação entre profissional e criança^(4)^. Nessa conjuntura, observa-se que o profissional de saúde precisa de habilidades internas e de ferramentas de comunicação para uma interação efetiva. As habilidades de comunicação são iniciadas ainda na formação do profissional e lapidadas na prática, enquanto as ferramentas, frequentemente, são desenvolvidas em um serviço e acabam se restringindo ao local de trabalho.

Como forma de ampliar as ferramentas comunicacionais na pediatria, a literatura vem crescendo em exemplos de estratégias que usam a tecnologia como mediadora em diversas especialidades pediátricas^(6-8)^. Alguns profissionais se valem de tecnologias leves, como cartilhas, fantoches^(9)^, brinquedos terapêuticos^(10)^, entre outros. No entanto, a literatura é escassa acerca de casos e estratégias específicas da urologia pediátrica fundamentadas em referenciais teóricos sólidos, com o uso comum de estratégias de forma empírica. Tais práticas são pouco disseminadas e, em diversos casos, compartilhadas apenas no serviço de saúde que os profissionais atuam.

## OBJETIVOS

Descrever as estratégias comunicacionais utilizadas por profissionais especialistas em urologia pediátrica para captar informações diretamente da criança.

## MÉTODOS

### Aspectos éticos

O estudo foi conduzido conforme diretrizes éticas nacionais e internacionais, sendo aprovado pelo Comitê de Ética em Pesquisa da Faculdade de Ciências da Saúde da Universidade de Brasília. Considerando as normas éticas para pesquisas que envolvem seres humanos, a aplicação do instrumento semiestruturado foi precedida da assinatura do Termo de Consentimento Livre e Esclarecido e do Termo de Autorização para Utilização de Imagem e Som de Voz, para fins de pesquisa. Em relação à identificação dos diferentes participantes, utilizou-se a denominação de PF1, PF2... PF14, com o intuito de preservar o anonimato dos profissionais.

### Referencial teórico-metodológico e tipo de estudo

Trata-se de estudo descritivo, de abordagem qualitativa, realizado com profissionais especialistas/*experts* que atuam na área de urologia pediátrica em Brasília. O estudo integra uma pesquisa de tese de doutoramento intitulada “Experiência de crianças com sintomas urinários e intestinais: contando essa história”^(11)^. A descrição metodológica seguiu o *checklist COnsolidated criteria for REporting Qualitative research* ^(12)^.

### Cenário do estudo

A pesquisa foi desenvolvida no Distrito Federal (DF), na unidade ambulatorial de um hospital público de ensino, além de outras duas unidades de saúde - uma privada e outra pública do Sistema Único de Saúde do DF -, com o objetivo de assegurar a participação de uma diversidade de profissionais especializados.

### Fonte de dados

A amostra foi de conveniência, e se valeu da técnica de bola de neve para indicação de profissionais. A amostra foi selecionada a partir da inclusão de profissionais com *expertise* na área de urologia pediátrica e que tinham experiência de, pelo menos, um ano no atendimento de crianças com sintomas urinários e/ou intestinais. O quantitativo de profissionais que compuseram a amostra seguiu o critério de saturação de dados^(13)^. O processo foi monitorado durante a análise temática, por meio da comparação contínua entre entrevistas e do acompanhamento da matriz de codificação. A saturação foi considerada alcançada quando as falas passaram a apresentar recorrência de sentidos e não emergiram novos elementos conceituais. A decisão final foi validada em consenso entre as pesquisadoras responsáveis pela análise.

### Coleta e organização dos dados

A coleta de dados ocorreu no período de fevereiro a maio de 2019. Os dados foram coletados por uma pesquisadora, acadêmica de enfermagem, previamente treinada quanto às técnicas de entrevista. A conversa com os entrevistados aconteceu no próprio ambiente de trabalho dos profissionais e em horário marcado, para que não houvesse impacto na rotina laboral. As perguntas norteadoras foram: quando você investiga os sintomas urinários e intestinais, é comum que você entreviste/converse com a criança, o responsável ou os dois? Como você faz essa investigação? Quais estratégias de comunicação você utiliza com a criança?

As entrevistas individuais tiveram duração média de 12 minutos. Entre os 14 profissionais abordados e convidados para participar da pesquisa, todos aceitaram prontamente. Não houve recusas, sendo informados sobre o objetivo da pesquisa no momento da abordagem inicial.

### Análise dos dados

As entrevistas foram gravadas em áudio e transcritas, sendo analisadas por meio do programa NVIVO, versão 12.0. Também foram realizadas anotações de campo após cada uma das entrevistas, e não foi necessário recontactar qualquer profissional para confirmação ou clarificação do conteúdo analisado.

O método de análise dos dados seguiu as etapas propostas por Braun e Clarke^(14)^, que incluía as seguintes etapas: familiarização de dados, por meio da transcrição e leitura exaustiva das entrevistas; produção de códigos iniciais; exploração de temas; revisão de temas; definição e denominação de temas; e produção do relatório. Todas as etapas foram realizadas com o auxílio do programa NVIVO e com a verificação de consenso entre as pesquisadoras envolvidas no processo de análise de dados.

## RESULTADOS

A amostra foi composta por 14 profissionais especialistas das seguintes categorias: enfermeiros (n=4); médicos (n=5); e fisioterapeutas (n=5). Tempo de experiência profissional foi, em média, de sete anos e 11 meses (dois a 25 anos). Todos os enfermeiros estavam no momento matriculados em cursos de pós-graduação, mestrado e/ou residência na área da enfermagem pediátrica, especificamente com atuação em urologia ou nefrologia pediátrica. No caso dos médicos, todos completaram a residência em nefrologia pediátrica. Os fisioterapeutas possuíam especializações em disfunções pélvicas, reabilitação do assoalho pélvico, fisioterapia uroginecológica, fisioterapia pélvica, ou tinham obtido mestrado e/ou doutorado em ciências médicas com trabalhos defendidos na área.

Os dados provenientes da entrevista foram analisados e codificados, compondo-se, primariamente, a matriz de codificação (Figura 1). Tal matriz ilustra o processo inicial de organização dos dados, em que os códigos emergiram das falas dos profissionais. A tonalidade indica a frequência de ocorrência dos códigos na entrevista, em que os tons mais escuros sinalizam maior recorrência.

**Figura 1:** Matriz de codificação dos dados, Brasília, Distrito Federal, Brasil

A árvore temática (Figura 2) apresenta a consolidação dos códigos em três categorias principais: Comunicação verbal; Comunicação não verbal; e Estratégias lúdicas. O diagrama evidencia como as categorias se inter-relacionam, demonstrando que a comunicação não verbal frequentemente complementa a verbal, enquanto o brincar integra elementos de ambas, configurando-se como estratégia híbrida. Essa representação visual permite compreender o percurso analítico, desde a codificação inicial até a formação de categorias que refletem práticas concretas de interação com a criança no contexto de urologia pediátrica.

**Figura 2:** Árvore temática. Brasília, Distrito Federal, Brasil

### Comunicando-se de maneira verbal com a criança

A comunicação verbal é o formato de linguagem descrito nessa categoria temática. Os profissionais de saúde entrevistados descrevem como conduzem seus atendimentos e dividem suas percepções entre a conversa direcionada aos responsáveis/cuidadores primários e diretamente à criança.

O diálogo com os responsáveis/cuidadores se mostra como a forma mais tradicional e confortável tanto para o profissional quanto para os próprios responsáveis/cuidadores primários. Notou-se, de forma comum, que os cuidadores assumem o diálogo e impedem o protagonismo da criança. Trata-se de uma forma de captação de informações de saúde sobre a criança atendida frequentemente utilizada.

*Muitas vezes, as mães nem deixam as crianças falarem, mas eu tento primeiro pegar alguma coisa com as crianças. As mães complementam - é claro que elas vão dar mais informações, mas eu tento tirar alguma coisa, alguma informação*. (PF1)

Os profissionais também exemplificaram como conduzir conversas diretamente com a criança. A principal estratégia por eles adotada é o emprego de linguagem e termos simples para tratar dos assuntos de saúde, além do uso frequente de termos no diminutivo, a fim de adequar a mensagem a ser transmitida ao nível de compreensão da criança. Analogias também foram implementadas nos diálogos, como associar a bexiga à “casa do xixi”.

*A principal é a questão da comunicação, é você sentar com a criança e tentar uma comunicação efetiva* [...] *você tá disposto ali pra escutar ela e tentar falar na linguagem que ela compreenda. Você tem que se fazer entender pela criança pra poder captar dela esses sintomas.* (PF6)

*É* [...] *eu falo diretamente com elas* [as crianças], *até pra consulta não ficar chata, não ficar achando que é uma coisa que a mãe que tem que chegar e falar comigo* [...] *eu tento usar uma linguagem mais simples, e mesmo sendo criança pequena, depois que o pai me relatou, eu sempre torno e pergunto à criança de novo.* (PF7)

*Manter um linguajar mais adequado também pra criança não utilizar termos muito mais difíceis* [...] *que ela entenda. A gente utiliza “xixi” e “cocô” mesmo, “dorzinha na barriga”, “saiu correndo”, “o xixi molhou a calcinha”.* (PF3)

*Então, as expressões que a gente usa, eu sempre trago “bexiga” e “intestino”, porque eu preciso falar que é a casa do xixi, a casa do cocô. E não trato de canal anal e uretra, eu vou tratar de “portinha”, a portinha do intestino, a portinha da bexiga.* (PF10)

Os profissionais também faziam uso de analogias com o cotidiano da criança para condução da conversa, aproximando o ouvinte da mensagem a ser transmitida.

*Então, a criança pergunta: “Por que que eu tenho que beber tanta água”, e aí você poderia explicar todo o funcionamento fisiológico do corpo e tudo mais, ou você poderia dar um exemplo mais simples, uma analogia bem mais simples que eles costumam entender bastante. Então: “Você não banha todo o dia?”, “você não lava seu corpo todo o dia?”. Então, você tem que lavar seu corpo por dentro também. Pronto, aí eles conseguem entender, fica bem mais fácil, bem mais tranquilo.* (PF4)

*É* [...] *eu tento trazer pra realidade deles perguntando dessa questão* [...] *“Você vai dormir na casa de um amiguinho e faz xixi na cama, você fica com vergonha?”, “Você vai na casa do seu amiguinho ou você não vai?”, pra ver o quanto isso atrapalha na parte social da criança.* (PF2)

*Eu sempre tento trazer pra eles o que que eles podem* [...] *a forma que eles podem entender* [...] *dentro da rotina deles, né* [...] *daquilo que eles têm condições de entender.* (PF5)

Ferramentas validadas de diagnóstico clínico são estratégias também apontadas pelos entrevistados como aliadas ao processo de comunicação. A Escala de Fezes de Bristol e o diário miccional guiaram os diálogos e captaram informações tanto com a criança quanto com os responsáveis/cuidadores primários.

*Eu geralmente mostro a Escala de Bristol. As crianças riem e às vezes têm vergonha, ou eu pergunto: “Qual é a cara do seu cocô? aí mostro a foto”.* (PF1)

*Eu geralmente pego no computador aquela Escala de Bristol, né, das fezes.* (PF14)

*Então, aí a gente usa aqui* [...] *é* [...] *a gente tem um diariozinho, né* [...] *a gente faz diário. Faz alguns questionários de qualidade de vida pra gente ver se a gente consegue* [...] *é* [...] *captar melhor a informação do paciente.* (PF8)

Assim, eu sempre tento trazer pra elas o que que vai acontecer dentro da realidade dela. “Então, a gente vai fazer esse diário aqui, mas esse diário é pra saber se você tá fazendo xixi na hora certa, então esse relógio que a gente vai usar é pra ele te explicar pra você *não se esquecer na hora que você tem que fazer o xixi”.* (PF5)

### Comunicando-se de maneira não verbal com a criança

Diferentemente da primeira categoria temática, aqui são abordadas estratégias que se valem da linguagem não verbal como norteadora da comunicação com a criança. Imagens e desenhos foram as estratégias mais citadas por profissionais nesse eixo. O uso se pautou nos princípios de emprego do lúdico na comunicação, além de buscar a materialização de exemplos, tornando-os mais concretos ao nível de pensamento da criança. Os profissionais usaram tal estratégia tanto para a expressão de sentimentos e emoções quanto para a representação física do corpo humano, que favorece a localização de sintomas e exemplificação mais concreta de posicionamentos, por exemplo.

*Eu utilizo de desenho, da bexiga, dos rins, dos ureteres, né, pra poder explicar que enche e como que esvazia a bexiga.* (PF3)

*Tentar ser o mais visual possível* [...] *é* [...] *mostrar uma imagem. Então, eu tenho uma revistinha que eu levo na minha pasta comigo. Ele vai mostrar a parte do abdômen, e aí, lá na parte do abdômen, a gente, aí, eu começo falando: “Ah, se você tirar essa parte da pele, é isso que você vai ver”, “Se você pegar, olha, pega aqui”, “Você sabe onde você, quando você sente vontade de fazer xixi, onde é que dói?”. Aí eles apontam, né? Apontam. Eles sabem onde fica a bexiga. “Então, a bexiga é isso, é um balãozinho tá aqui”.* (PF4)

*Eu sempre trago, às vezes, tipo um desenho, e peço pra elas desenharem como que elas se sentem, pra que elas entendam o que tá acontecendo, e aí eu mostro através dos desenhos mesmo. “Ó, no caso, você tem um probleminha aqui”.* (PF5)

*E desenhos, muitos desenhos, mostrando pra ela* [...] *materializando aquela informação pra criança. Como ela é muito visual e lúdica, então a gente tenta sempre mostrar alguma coisa pra ela tentar associar aquela informação àquele objeto que ela viu.* (PF9)

*Mostro também no* [...] *uso o computador aquelas técnicas de compressão, de agachamento, pra mostrar pros pais e pra criança, pra ver se eles identificam como um comportamento deles, né?* (PF14)

Outros exemplos de ferramentas utilizadas são os *folders*, revistas e livros. Os profissionais afirmam que são materiais com informações importantes sobre o tratamento, apresentando imagens que apoiam na hora da exemplificação das orientações clínicas e permitindo a identificação da criança com sua própria experiência de sintomas. Assim, tais recursos de educação em saúde também exercem papel significativo na comunicação com a criança.

*Eu utilizo folder pra que eles possam ver também, né, através de imagens e desenhos, as cartilhazinhas - mais direcionadas para os pais, que eles vão ler, e a criança que já sabe ler a gente já entrega pra criança.* (PF3)

*Se é uma criança muito difícil, não tá sabendo realmente falar, reportar os sintomas dele. A gente usa muito livros educativos, principalmente aquelas criancinhas que estão na época de desfralde, essas coisas assim.* (PF8)

*Eu tenho uma revistinha que eu levo na minha pasta comigo. Ele vai mostrar a parte do abdômen.* (PF4)

*Eu utilizo livros relacionados aos temas.* (PF11)

*Também tenho uma revistinha que mostra que a criança tem, né* [...] *que é comum, né, que a criança perca urina dormindo, que isso não é um probleminha só dela; pode ser de várias crianças. É uma revista lúdica que eu trago pra eles e mostro como que é importante fazer o tratamento certinho, pra que eles possam ter os resultados e melhorar em relação a isso.* (PF5)

De maneira menos frequente nas entrevistas, também foram apontados maquetes, vídeos e objetos do cotidiano. Todos possuem a finalidade de tornar mais tangível a informação, dentro de uma perspectiva de mensagem concreta.

*A gente tem lá uma maquete dinâmica que, quando eu vou explicar o sistema urinário, eu utilizo ela, que ela é bem legal, bem grande assim* [gesticula]; *tem todo o sistema urinário. Então, eu vou explicando pra eles* [...] *e isso prende a atenção deles.* (PF9)

*Pra poder eles verem, visualizarem, eu utilizo vídeo também.* (PF3)

*Eu utilizo copo pra fazer o barulho do xixi. Enfim, tudo o que a gente tem de lúdico, né* [...] (PF11)

Até mesmo os aparelhos de terapia por neuromodulação, como *biofeedbacks*, são adaptados com imagens para auxiliar na comunicação com a criança.

*Enfim, tudo o que a gente tem de lúdico, né* [...] *o biofeedback lúdico, a gente utiliza, né.* (PF11)

### O brincar e o lúdico na comunicação com a criança

Essa categoria inclui o brincar e o lúdico como ferramentas de comunicação. Trata-se, portanto, de uma categoria que abarca elementos verbais e não verbais, ou seja, peças fundamentais do brincar, sendo uma categoria que se relaciona com as duas primeiras.

O uso de bonecos foi uma das estratégias de brincar adotadas. O boneco permite a demonstração dos sistemas urinário e intestinal, e a identificação da criança com os sintomas que apresenta. Além disso, os profissionais usam o boneco para ilustrar orientações clínicas, como posicionamento no vaso sanitário. Deve-se lembrar que não são necessariamente bonecos específicos de urologia, mas muitas vezes adaptados para as orientações e contexto, sendo o feltro um exemplo de material utilizado para a construção de bonecos.

*Quando eu vou, por exemplo, é* [...] *mostrar posicionamento correto de toalete, a gente tem dois bonecos lá grande, de pano - a menina e o menino. Quando é uma menina, eu pego uma boneca menina e tento mostrar pra ela, fazendo a boneca se posicionar certinho* [...] *“Ó, seus pés têm que tá rente ao chão, né, pra você relaxar o assoalho pélvico”* [...] *e vou explicando* [...] *lógico que na linguagem da criança.* (PF9)

*A gente tem um boneco de feltro que ele tem bexiga e intestino, porque, às vezes, quando eu trato essas expressões, a criança de 6 a 12 anos, isso é mais fácil quando eu falo de bexiga e de intestino*. (PF10)

O ato de contar histórias também foi uma estratégia lúdica adotada. Os profissionais faziam uso da dramatização e demonstração para que houvesse compreensão direta da criança sobre a orientação clínica.

*Eu faço teatro. Eu mesmo, mostrando que a criança tem que abaixar, principalmente a menina, abrir a perna, apoiar o pé, aí eu ensino que tem que sentar no vaso de perna* [...] *com os joelhos bem afastados. Quando, principalmente, as meninas, esquecem, etc., eu falo que tem que sentar ao contrário no vaso por causa do formato do sanitário. Se você entrar de frente, olhando pra descarga, não tem jeito de você não abrir as pernas.* (PF14)

Os jogos e brinquedos também foram aliados no processo de comunicação, especialmente para o reforço de orientações e captação de sintomas experienciados pela criança. Importante frisar que também são produzidos pelos próprios profissionais, uma vez que não se dispõe de vastas opções no mercado.

*Eu tenho uns joguinhos de memória que às vezes eu mostro pra elas.* (PF5)

*A gente utiliza alguns brinquedos, alguns joguinhos, pra eles tentarem colocar esses sintomas deles.* (PF6)

## DISCUSSÃO

O modo verbal é a forma mais tradicional de comunicação, não sendo distinto no contexto de atendimento da urologia pediátrica. Apesar de os atendimentos serem majoritariamente direcionados aos responsáveis^(4)^, os entrevistados trouxeram sugestões de condução da comunicação direta com a criança. Pautaram-se nos conhecimentos sobre desenvolvimento infantil e demonstraram que a conversa deve ser adequada ao nível desenvolvimental da criança. Adaptam-se à linguagem, buscando-se semelhanças com o cotidiano para que o assunto abordado se torne mais palatável. Os assuntos a serem abordados pelos profissionais podem ser complexos, como a desmistificação sobre o sistema urinário e intestinal como parte da uroterapia^(15)^.

A adaptação da linguagem, como o uso de termos simples e o emprego de diminutivos, mostrou-se essencial para aproximar o profissional do universo cognitivo e emocional da criança. Tal abordagem dialoga com o modelo teórico de Piaget, que descreve o estágio operacional concreto (7 a 11 anos) como um período em que o pensamento infantil é predominantemente concreto e baseado em experiências diretas com a realidade^(16)^. Nesse contexto, o pensamento simbólico da criança é caracterizado pela utilização de imagens e representações concretas de sua vivência cotidiana^(17)^. Ao empregar uma comunicação que ressoe com a perspectiva cognitiva da criança, os profissionais não apenas facilitam a compreensão, mas também criam um ambiente mais acolhedor e seguro, favorecendo a expressão espontânea e o engajamento ativo da criança no diálogo.

Considerando que os sintomas urinários e intestinais são mais frequentes em escolares^(1,18)^, sob a ótica de Piaget, justifica-se que as estratégias comunicacionais sejam pautadas fundamentalmente no pensamento concreto, tanto nas técnicas verbais quanto nas não verbais. O uso de imagens, vídeos, cartilhas, entre outros, foi citado como forma de materializar as orientações dadas pelos profissionais, uma vez que precisam trabalhar com a informação e desmistificação sobre os sintomas e os sistemas que compõem a micção e evacuação, promovendo a modificação de hábitos miccionais e de evacuação - elementos da uroterapia^(2,15)^. Experiências semelhantes em enfermagem pediátrica demonstram que o uso de materiais visuais e de dramatizações facilita a compreensão de processos fisiológicos, estimulando o protagonismo infantil no cuidado^(19,20)^. Assim, o emprego de recursos concretos e lúdicos contribui para a efetividade das intervenções educativas em urologia pediátrica, reforçando o papel da enfermagem na mediação do aprendizado e da adesão terapêutica.

Tais métodos já são empregados em outras áreas da saúde^(21-23)^, como o uso de imagem para localização de dor^(24)^, demonstrando potencial de aplicação na urologia pediátrica, sobretudo no apoio à comunicação e educação em saúde. Estudo sobre o modo como a criança expressa seus interesses em internações ressalta o fornecimento de informações preparatórias para a tomada de decisão como sugestão, que inclui o uso de imagens que representem os procedimentos a que a criança será submetida^(25)^. Estudos internacionais, mapeados em revisão de escopo, sobre incontinência urinária pediátrica também evidenciam o potencial de ferramentas digitais e vídeos animados na educação da criança, com resultados semelhantes aos obtidos pela uroterapia padrão na redução dos sintomas urinários e intestinais^(26)^. Para a enfermagem, esses achados indicam oportunidades de desenvolver e validar recursos audiovisuais que integrem linguagem técnica e sensibilidade infantil, fortalecendo a prática educativa e a adesão terapêutica.

Abordagens baseadas em artes também se alinham aos apontamentos feitos pelos entrevistados, uma vez que os exemplos apresentados podem ser classificados em tipos de artes, tais como desenhos, vídeos, cartilhas (artes visuais), bonecos e dramatização (artes cênicas). As artes são amplamente utilizadas na interação com a criança pela pedagogia e psicologia^(27-29)^, em ações de educação na saúde^(19,20,22,23)^, e em pesquisas na pediatria para a coleta de dados precisos^(30-32)^.

O uso do lúdico e do boneco terapêutico alinha a linguagem verbal e a não verbal dentro de um contexto de história. A técnica de *storytelling* é uma forma de comunicação muito aceita pelas crianças, que conseguem, por meio dos neurônios-espelhos, projetar-se na história, compreendendo melhor as mensagens e exteriorizando sua própria vivência^(33)^. O boneco terapêutico de feltro citado pelos profissionais é usado majoritariamente para educação em saúde. Além disso, a literatura mostra outros usos do boneco, que incluem a expressão de sentimentos relativos ao tratamento, a simulação de procedimentos clínicos e o treinamento de funções fisiológicas. São notórios os benefícios desse tipo de técnica, apontados não somente pela equipe de saúde, mas também pelos familiares^(34)^.

Além do uso de bonecos, iniciativas que unem artes, história e tecnologia na urologia pediátrica são possíveis e se adequam à predileção atual das crianças por brincadeiras tecnológicas. A exemplo, pode-se citar recente protótipo de história digital voltado ao atendimento de tais crianças^(11)^. Outros exemplos de uso de tecnologia na uroterapia são os aplicativos. Apesar de não terem sido mencionados pelos entrevistados, é possível identificar para as incontinências^(35,36)^, incluindo formatos que atuam como intervenção terapêutica pelos lembretes que fornecem ao paciente e como ferramenta de educação em saúde^(36)^, assim como aplicativos que auxiliam no acompanhamento das frequências urinárias e favorecem o preenchimento do diário miccional^(37)^. Apesar das múltiplas opções de aplicativos, poucas são destinadas ao público infantil.

### Limitações do estudo

Podem ser consideradas duas limitações principais do estudo. A primeira diz respeito à regionalização da coleta de dados, uma vez que a comunicação também sofre impacto da região e cultura. A segunda se relaciona ao cenário de trabalho. Como os profissionais foram indicados pela técnica de bola de neve, houve predominância de três serviços de saúde. Os enfermeiros, por exemplo, foram capacitados no mesmo ambulatório, e, por isso, as estratégias apontadas pelas entrevistadas eram semelhantes.

Desse modo, estudos futuros devem ampliar o número de serviços de saúde e expandir a coleta para outros estados brasileiros, a fim de identificar estratégias comunicacionais específicas e regionais. Apesar das limitações, Brasília é uma cidade multicultural e recebeu historicamente muitos migrantes das diferentes regiões, o que pode ter minimizado as limitações do estudo. Os serviços que os profissionais entrevistados trabalham são de grande volume de atendimento na cidade, podendo refletir um panorama ampliado. Além disso, o desenho qualitativo permite um olhar aprofundado para as estratégias comunicacionais, explorando-se o fenômeno estudado com maior profundidade.

### Contribuições para área da enfermagem, saúde ou políticas públicas

As conclusões deste estudo evidenciam a necessidade crítica de aprimorar as competências comunicacionais dos profissionais de saúde no campo da urologia pediátrica. A formação e a educação permanente dos profissionais devem promover o desenvolvimento de habilidades específicas para interagir de forma eficaz com a criança e sua família, especialmente em contextos de intervenções complexas. Essa capacitação deve abranger não apenas a comunicação verbal, mas também a utilização de estratégias não verbais e lúdicas que favorecem a compreensão, a adesão terapêutica e o vínculo profissional-criança e família.

Para a prática clínica, sugere-se a importância de integrar, de forma sistemática, abordagens que combinem essas três categorias de comunicação no atendimento de crianças em urologia pediátrica. A implementação dessas estratégias, geralmente de baixo custo e fácil aplicação, pode melhorar significativamente a qualidade do cuidado e a experiência da criança no ambiente de saúde.

## CONSIDERAÇÕES FINAIS

Diferentes estratégias comunicacionais de captação de informações diretamente da criança foram apresentadas pelos profissionais entrevistados e especialistas em urologia pediátrica. Foi possível classificar as estratégias em verbais, não verbais e brincar/lúdico. No entanto, evidencia-se que os profissionais não utilizam apenas um tipo de estratégia, sendo comum a associação das três categorias em um único atendimento.

Desse modo, adaptação da linguagem, foto-elicitação e contextualização das orientações com ferramentas lúdicas se mostram como modelos efetivos de comunicação dentro da urologia pediátrica. Tais achados oferecem subsídios para construção de instrumentos de educação em saúde urológica, elaboração de protocolos comunicacionais específicos para a pediatria e planejamento de ações de capacitação profissional voltadas para o desenvolvimento de competências comunicacionais.

Desse modo, o estudo traz exemplos de estratégias de baixo custo e de fácil implementação no cotidiano de atendimento, contribuindo para qualificação da interação entre profissional e criança em urologia pediátrica, promovendo maior adesão ao tratamento e potencial melhora dos desfechos clínicos. Além disso, evidencia oportunidades para o desenvolvimento e validação de novas ferramentas comunicacionais em pesquisas futuras que possam ser amplamente incorporadas à prática clínica e avaliadas por meio de estudos longitudinais.

## Data Availability

Os dados de pesquisa estão disponíveis no corpo do artigo.
